# MetaboReport: from metabolomics data analysis to comprehensive reporting

**DOI:** 10.1093/bioinformatics/btae373

**Published:** 2024-06-17

**Authors:** Yonghui Dong, Sergey Malitsky

**Affiliations:** FAFU-UCR Joint Center for Horticultural Biology and Metabolomics, Haixia Institute of Science and Technology, Fujian Agriculture and Forestry University, Fuzhou, 350002, China; Life Science Core Facilities, Weizmann Institute of Science, Rehovot, 76100, Israel; Life Science Core Facilities, Weizmann Institute of Science, Rehovot, 76100, Israel

## Abstract

**Motivation:**

Metabolomics, as an essential tool in systems biology, is now widely accessible to researchers of all levels. Yet challenges remain in data analysis and result interpretation. To address these challenges, we introduced MetaboReport, a versatile and interactive web app that simplifies metabolomics experiment design, data preprocessing, exploration, statistical analysis, visualization, and reporting.

**Results:**

MetaboReport produces a comprehensive HTML report, including project details, an introduction, interactive plots and tables, statistical results and an in-depth explanations and interpretation of the results. MetaboReport is particularly tailored for research labs and metabolomics core facilities that provide metabolomics services, allowing them to efficiently manage and document different metabolomics projects, and effectively report the metabolomics results to users.

**Availability and implementation:**

MetaboReport is freely accessible on https://metaboreport.com, with source code available on GitHub (https://github.com/YonghuiDong/MetReport). Alternatively, users can install MetaboReport as a standalone desktop app (https://metaboreport.sourceforge.io).

## 1 Introduction

Metabolomics, which leverages cutting-edge analytical chemistry techniques and advanced statistical methods to characterize the complete set of metabolites present in biological systems, has become an essential tool for systems biology (Wishart 2016). Many of these metabolomics platforms are situated within institutional and/or national core facilities, providing a range of metabolomics capabilities to researchers (Hunter *et al.* 2017). Metabolomics is no longer described as novel concept; it is now easily accessible to researchers at all experience levels. A number of software have been developed for metabolomics data analysis, including the proprietary tools like Compound Discoverer (Thermo), Progenesis QI (Waters), MetaboScape (Bruker), and open-source solutions such as XCMS (Smith *et al.* 2006), MS-DIAL (Tsugawa *et al.* 2020), MZmine (Schmid *et al.* 2023), and MetaboAnalyst (Pang *et al.* 2022, 2024). All these tools offer great convenience, allowing users with limited background in metabolomics and statistics to perform data analysis effortlessly. However, the metabolomics data is difficult to translate into biologically relevant results and the statistical results could be hard to understand and interpret, particularly for those new to the field.

There is a clear need for user-friendly tools that streamline data preprocessing, statistical analysis, visualization, and notably, the interpretation and elucidation of results via an intuitive and guided approach. To this end, we have developed MetaboReport, a web-based application, to help users load, explore, analyze, and visualize their data in a simple and intuitive way. The resulting HTML-format report can be used to manage, track, and archive the metabolomics projects, and particularly, to assist users in understanding and interpreting their metabolomics results.

## 2 Materials and methods

**Figure 1.**
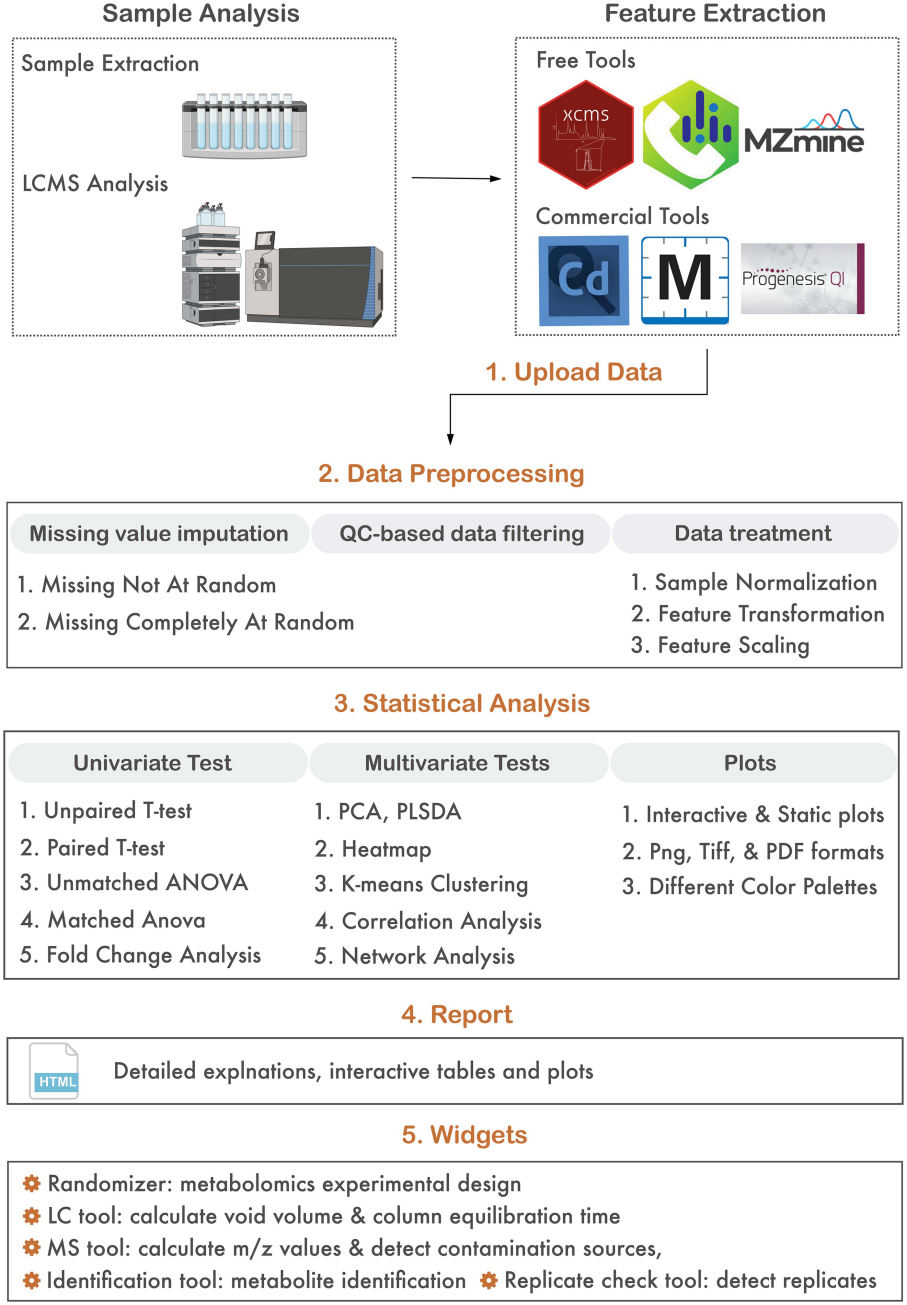
Representative workflow and major functions of MetaboReport.

MetaboReport is an R Shiny app (Beeley and Sukhdeve 2018) written in a modular way following the golem schema for production grade app development (Fay *et al.* 2021). This approach allows MetaboReport to be distributed as a regular R package, which is fully cross-platform and can be launched locally from any computer that has R installed. In addition, MetaboReport is hosted by the shinyapps.io server (https://www.metaboreport.com), and available as a standalone desktop app (https://metaboreport.sourceforge.io). This option offers significant convenience as users are not obligated to install R or any associated packages. Moreover, the encapsulation of all necessary components within the web app or standalone desktop application ensures a more stable and consistent performance regardless of variations in the user's local environment or system configurations. MetaboReport includes five major sections, data uploading, preprocessing, statistical analysis, report generation, and a collection of widgets (Fig. 1).

### 2.1 Data uploading

MetaboReport supports a diverse range of outputs generated by various software tools, such as XCMS, MSDIAL, mzMine, Compound Discoverer and Progenesis QI (Fig. 1). After feature extraction with proprietary or open-source software, users can directly submit the resulting mass feature table (feature in rows and sample in columns) to MetaboReport. Both comma-separated value (csv) and Excel (xls or xlsx) formats are supported (Fig. 1). Sample meta information can be included in the sample column names in the feature table, or alternately, users can provide a separate metadata table. For more information on the data table preparation, please refer to the user manual (Supplementary Information S1) and demo data (Supplementary Information S2 and S3).

### 2.2 Preprocessing

The objective of data preprocessing is to generate “clean” data for subsequent statistical analysis (Van Den Berg *et al.* 2006). Data preprocessing encompasses missing value imputation, quality control (QC)-based data filtering, and data treatment, including normalization, transformation, and scaling (Fig. 1). MetaboReport employs five missing value imputation methods, single value imputation (1, 1/5 minimum, mean, median) and k-nearest neighbor (KNN), to handle missing values arising from different mechanisms such as missing not at random and missing at random (Wei *et al.* 2018). When pooled QC samples are available, MetaboReport empowers users to identify and exclude unstable mass features through the application of a predefined relative standard deviation (RSD) threshold. Log10-transformation is applied to address heteroscedasticity and symmetrize skewed distributions. To reduce unwanted sample-to-sample variation, five normalization methods are implemented in MetaboReport, including internal standard-based normalization, linear baseline normalization based on mean or median values, probabilistic quotient normalization, and quantile normalization (Ejigu *et al.* 2013). Lastly, MetaboReport provides six data scaling approaches, mean centering, auto scaling, pareto scaling, range scaling, vast scaling, and level scaling, to ensure that all mass features are aligned on a comparable scale (Van Den Berg *et al.* 2006). One distinct feature of MetaboReport is the use of several different interactive plots, such as total ion current boxplot, principal component (PCA) score plot, and mass feature density plot and boxplot to help users choose suitable data preprocessing methods. These plots update dynamically as the data preprocessing methods change, thereby enhancing method selection effectively and seamlessly.

### 2.3 Statistics

MetaboReport offers a wide variety of univariate and multivariate analyses, including t-test, ANOVA, fold-change analysis using paired and unpaired samples, PCA, partial least squares-discriminant analysis (PLS-DA), clustering analysis, correlation analysis, and network analysis (Fig. 1). Both interactive and static figures are provided for each analysis. Interactive figures facilitate the dynamic exploration of specific data points or regions of interest; and static figures are high-resolution publication-ready plots, which can be downloaded in various formats (i.e. PDF, PNG, and TIFF) with different color palettes and customable aspect ratio. Another unique feature of MetaboReport is that it allows users to meticulously curate their datasets by selectively excluding or including specific samples (e.g. outliers) and distinct sample groups. This fine-tuned control ensures that the statistical analysis and visualization precisely align with the user's objectives. Furthermore, MetaboReport facilitates seamless exploration of multiple sets of metadata (if available). This functionality enables users to effortlessly switch between different metadata sets for each analysis, facilitating a comprehensive understanding of their data from different perspectives.

### 2.4 Report generation

After statistical analyses, users can export a comprehensive HTML report. This report includes essential project details such as the project name, lab/author names, and date. It comprises an introduction, all previously mentioned interactive plots and tables, summary statistics, and detailed explanations. Furthermore, this report can be used for individual labs or core facilities to manage, track, report, and archive metabolomics projects.

## 3 Results

The use of MetaboReport is showcased with three datasets. Dataset 1 is from a LC-MS-based spatial metabolomics study. These datasets consists of 94 samples collected from 14 distinct lamprey tissues, analyzed at negative ion modes (Supplementary Information File S4) (Gou *et al.* 2023). The PCA score plot generated by MetaboReport clearly shows that the buccal gland is distinctly separated from all other tissues, indicating a unique metabolic profile. The hierarchical clustering heatmap further underscores this point, revealing that a significant proportion of features are enriched in the buccal gland. Further statistical analysis reveals a remarkable accumulation of metabolites in this gland. Specifically, 182 mass features were observed to be over 1000-times higher in the buccal gland compared to the other 13 tissues, with a statistically significant FDR-adjusted *P*-value of <0.001. Notably, two specific groups of metabolites, prostaglandins and kynurenine pathway metabolites, were exclusively detected in the lamprey buccal gland. This suggests that the buccal gland may serve as a repository of pharmacologically active components that modulate host homeostasis, inflammatory, and immune responses. In addition, MetaboReport is accompanied by a demo LC-MS dataset to assist users becoming familiar with the workflow. The demo data can be loaded by simply clicking the load demo data button in the data upload section. The resulting reports are shown in File S5.

A number of web tools have been developed for metabolomics data analysis. Among them, MetaboAnalyst 6.0 (Pang *et al.* 2024) is the most comprehensive one. As such, we have compared the main features of MetaboReport with those of MetaboAnalyst 6.0 (File S6). Indeed, MetaboAnalyst 6.0 offers a wide range of functionalities that cover various aspects of metabolomics research, such as raw data processing, data preprocessing, statistical analysis, functional interpretation, and results visualization. On the other hand, MetaboReport offers a more focused set of features that may complement those of MetaboAnalyst 6.0 (File S6). For instance, it provides different widgets that facilitate metabolomics experiment design and studies. The generated HTML report contains more detailed explanations than those provided by MetaboAnalyst, thereby aiding users in understanding and interpreting their metabolomics results.

## 4 Conclusion

MetaboReport is an interactive web-based tool designed to streamline the exploration, statistical analysis, visualization, and reporting metabolomics data. It offers a structured and user-friendly workflow, making it a flexible tool for users of all levels. It is particularly well-suited for research labs and metabolomics core facilities that offer metabolomics services, enabling them to effectively manage, track, report, and archive different metabolomics projects.

## Supplementary Material

btae373_Supplementary_Data

## Data Availability

All data used for the analyses in this article are publicly available. The lamprey metabolomics dataset has been archived in MetaboLights with the identifier of MTBLS5857.
