# Supplementary material for: MetaboReport: from metabolomics data analysis to comprehensive reporting
Source: Bioinformatics. 2024 Jun 17;40(6):btae373. doi: 10.1093/bioinformatics/btae373 (PMC11209541; doi:10.1093/bioinformatics/btae373)
Supplement: btae373_Supplementary_Data [file btae373_supplementary_data.zip › FileS5_Report_Lamprey_Neg.html]

Report for Project: Lamprey\_Neg


# Report for Project: Lamprey\_Neg

#### Metabolic Profiling Unit, Weizmann Institute of Science

#### 01, February, 2024

# 1 Introduction

Metabolomics involves the comprehensive analysis of
low-molecular-weight metabolites in biological systems. To address
specific research questions or applications, there are three widely-used
strategies in metabolomics [1]:

`Untargeted Assay`: (or frequently referred to as
untargeted metabolomics or global metabolomics). Its objective is to
reproducibly measure as many metabolites as feasible, and provide
semi-quantitative data (chromatographic peak areas are reported, not
concentrations). The chemical identity of metabolites is not necessarily
known prior to data acquisition.

`Targeted Assay`: (or sometimes referred to as targeted
metabolomics). This approach focuses on a small number of metabolites of
interest whose chemical identity is known prior to data acquisition, and
an absolute concentration of each metabolite is typically reported.

`Semi-targeted Assay`: (or frequently referred to as
metabolic profiling). It acts as an intermediate between untargeted and
targeted analyses, where typically hundreds of metabolites are targeted,
whose chemical identity is known prior to data acquisition.
Semi-quantification information is reported in this type of
approach.

This report is an overview of your data analysis results. Brief
explanations are provided in each section to help you understand the
report. Below are few tips on how to use this report.

> **Tips:**
>
> 1. Most figures in this report are interative, which means you can
>    zoom and pan the figures, and hover over them to get more detailed
>    information. Don’t download figures from this report, and use them for
>    publication purpose. The figure resolution is too low (72 dpi).
> 2. High resolution (600 dpi) static figures are provided along with
>    this report. You can also download them in `Statistics` tab
>    if you are using **MetaboReport** software by yourself to
>    generate this report.
> 3. If you want to perform additional statistics and/or re-analyse
>    you data by yourself, you can use either Table 1 (raw data) or Table 2
>    (data with different statistical information). You can click
>    `Download` button above the table to download it in
>    **cvs** or **excel** format.

# 2 Results

## 2.1 Quality Control

> **Note:**
>
> If your project does not contain QC samples, you can skip this
> section.

Robust and reproducible data is essential to ensure high-quality
analytical results, and is particularly important for large-scale
metabolomics studies where detector sensitivity drifts, retention time
and mass accuracy shifts frequently occur. Therefore, raw data need to
be inspected before data processing to detect measurement bias and
verify system consistency [2].

The use of quality control (QC) is now routine to monitor, evaluate
and correct system variations in metabolomics studies. Different types
of QC samples are used in metabolomics, such as pooled QC samples,
reference materials, standard reference materials and long term
reference (LTR) samples.

### 2.1.1 PCA-based QC Evaluation

A rapid systematic check of data quality can be made by performing
principal components analysis (PCA) on the complete data set (For
explanation of PCA, please refer to **section 3.2.1**). By
plotting the first two principal components scores, and labeling the
data points as either QC samples or biological samples, the difference
in multivariate dispersion can be visually assessed.

Ideally, QC samples should be clustered tightly in comparison to the
total variance in the projection. If you used
`pooled QC samples` in your study, the QCs should cluster at
the center the PCA scores plot. Any deviation from the origin is usually
due to unavoidable pipetting errors or sample weight discrepancies, or
when the pooled QC is not generated from sub-aliquots of all the
biological test samples.

As long as the QCs cluster tightly, relative to the observed
dispersion of biological samples, then these data can be deemed as of
high quality [1].

### 2.1.2 Comprehensive QC Report

In addition, you may receive an HTML format QC report. It was
generated using R package **RawHummus**, which was designed
for quick and comprehensive evaluation of the metabolomics data quality
based on QC samples. `RawHummus` adopts 12 quality metrics
which are closely related to liquid chromatography (LC) peak shape,
retention time (RT), mass accuracy, detector sensitivity and
fragmentation to to comprehensively evaluate the raw data quality
[2].

> **Note:**
>
> Detailed evaluation of the data quality can be found in the RawHummus
> report.
>
> This RawHummus report can be direcrly used as a supplementary
> metarial in your publication.
>
> Please cite RawHummus publication in your manuscript [2].

## 2.2 Statistical Result

### 2.2.1 PCA

Principal Component Analysis (PCA) is an unsupervised data analysis
method, which transforms a set of correlated variables into a set of
linearly uncorrelated variables. The uncorrelated variables are ordered
in such a way that the first one accounts for as much of the variability
in the data as possible and each succeeding one has the highest variance
possible in the remaining variables. These ordered uncorrelated
variables are called `principle components`. By discarding
low-variance variables, PCA helps reduce data dimension and visualize
the data.

The PCA scores represent the new location of the samples in each
principal component. A typical way to look at these is to plot the
scores values in two dimensions, corresponding to pairs of components
(usually PC1 vs PC2). Each point in a scores plot therefore represents a
sample, with samples close together being more similar to each other,
and those further apart being more dissimilar. By coloring by sample
type, we can check, (i) the consistency of the QC samples (as discussed
above in section **3.1.1**), and (ii) the presence of any
sample outliers (any samples which are very different to the
others).

> **Note:**
>
> The PCA score plot is identifical as shown in section 3.1.1. The plot
> in section 3.1.1 is mainly used for data quanlity check, while here it
> is used to get an overview of the data and detect any sample
> outliers.

### 2.2.2 Clustered Heatmap

A clustered heatmap is a representation where values are represented
on a color scale. One can cluster samples and mass features to identify
groups of mass features that show a coordinated behavior.The rows of the
heatmap below represent samples, and the columns represent mass
features.

> **Note:**
>
> Depending on the parameter settings in the
> **MetaboReport** data analysis work, heatmap can be made
> from either all the mass features detected in the samples or only
> statistically significant mass features.
>
> 1. Heatmap constructed with all the mass features (or metabolites)
>    gives an overview of the data structure.
> 2. Heatmap constructed with only statistically significant mass
>    features (or metabolites) allows better visualization of the sample
>    group differences.

### 2.2.3 Data Overview


**Table 1. Data table with statistical information**

> **How to interpret Table
> 1?**
>
> **(1) Parameters in Table 1**
>
> `Fold_X_vs_Y`: Fold change (FC) between Group X and Y. FC
> is a measure describing how much a metabolite (or mass feature) changes
> between Group X and Y. It is defined as the ratio between X and Y, i.e.,
> X/Y.
>
> `AdjPvalue_X_vs_Y`: Adjusted P-value between Group X and
> Y. The adjusted p-value gives information on statistical significance
> between Group X and Y.
>
> `VIP_X_vs_Y`: Variable importance in projection between
> Group X and Y. It is a metric produced by Partial Least-Squares
> Discriminant Analysis (OPLS-DA). (for more information on OPLS-DA can be
> found in Figure 3).
>
> **(2) How to perform feature selection?**
>
> In metabolomics, feature selection is closely related to putative
> biomarker assessment, i.e., finding metabolites that are most relevant
> in discriminating group X and Y (for instance, wild type and
> mutant).
>
> You can use a multi-factor approach (i.e., `FC`,
> `adjusted p-value`, and `VIP`) to select potential
> biomarkers in your study based on the statistical results in Table
> 2.
>
> Our suggested values are: mass features with adjusted p-value <
> 0.05 and VIP > 1 are considered as statistically significantly
> different, and FC >= 2 as increase and FC <= 0.05 as decrease. See
> **Table 2** below.

---

### 2.2.4 Differential Features

**Table 2. Summary of Statistical Result**

The following criteria are used to select statistically significantly
different mass features (or metabolites):

The mass features with adjusted **p-value < 0.05**
and **VIP > 1** are considered as statistically
significantly different, and **FC >= 2** as increase and
**FC <= 0.05** as decrease.

Below you can find the detailed information of the differential mass
features in each compared groups.

### 2.2.5 PLS-DA

PLS-DA is a supervised method in which prior knowledge of grouping in
the data set is incorporated into the principal component calculations
in order to maximize group separation. This approach is excellent in
finding differential metabolites between two groups, but it has the
caveat of over-fitting the data, which will produce good looking plots
with nonsensical data. Therefore, PLS-DA data have to be thoroughly
tested for over-fitting by cross-validation and permutation tests.

PLS-DA data are generally reliable when R2Y values are close to, but
not equal or above, 1 and the Q2Y value above 0.4. Also, the results for
the permutation tests (pR2Y and pQ2Y) should be below 0.05 [3].

The `S-plot` is a visualization method that combines the
modeled covariance (X-axis) and modeled correlation (Y-axis) from the
PLS-DA on a scatter plot, allowing for pinpointing of interesting
variables. The variables showing the highest covariance and correlation
values with variable influence on projection (VIP) >= 1 are
considered the most relevant variables for the classification between
samples.

### 2.2.6 Volcano Plot

### 2.2.7 K-Means Cluster Analysis

K-Means Cluster Analysis can be used to reveal the main subsets of
mass features that displayed differential profiles across different
sample groups.

The `Cluster Plot` shows the trend of the metabolic
profiles in each subset (subgroup)

The `Table` provides more details information of each
metabolite, such as metabolite name (if exit), standardized peak area
and the group information.

**Metabolic Profile Clustering**

### 2.2.8 Barplot

The bar plot shows the quantitative information of each metabolite in
different sample group.

> **Note**
>
> Depending on the slected setting in **MetaboReport**
> workflow, different data transformation is used before maiking barplot,
> please refer to the y-axis legend for the data transformation
> method.
>
> In the selection box, you can choose the metabolite to display the
> plot for this metabolite. The corresponding table information of this
> selected metabolite will be displayed as well.

Select a metabolite

**Table 4: Metabolic information of selected
metabolite**

# 3 Reference

[1] Broadhurst, D., Goodacre, R., Reinke, S.N., Kuligowski, J.,
Wilson, I.D., Lewis, M.R. and Dunn, W.B., 2018. Guidelines and
considerations for the use of system suitability and quality control
samples in mass spectrometry assays applied in untargeted clinical
metabolomic studies. Metabolomics, 14(6), pp.1-17.

[2] Dong, Y., Kazachkova, Y., Gou, M., Morgan, L., Wachsman, T.,
Gazit, E. and Birkler, R.I.D., 2022. RawHummus: an R Shiny app for
automated raw data quality control in metabolomics. Bioinformatics,
38(7), pp.2072-2074.

[3] Westerhuis, J.A., Hoefsloot, H.C., Smit, S., Vis, D.J., Smilde,
A.K., van Velzen, E.J., van Duijnhoven, J.P. and van Dorsten, F.A.,
2008. Assessment of PLSDA cross validation. Metabolomics, 4(1),
pp.81-89.
