# Supplementary material for: MetaboReport: from metabolomics data analysis to comprehensive reporting
Source: Bioinformatics. 2024 Jun 17;40(6):btae373. doi: 10.1093/bioinformatics/btae373 (PMC11209541; doi:10.1093/bioinformatics/btae373)
Supplement: btae373_Supplementary_Data [file btae373_supplementary_data.zip › File S6_Comparison of MetaboReport with MetaboAnalyst 6.docx]

Table S7 Comparison of MetaboReport with MetaboAnalyst 6.0.

| **Functions** | **MetaboReport** | **MetaboAnalyst 6.0** |
| --- | --- | --- |
| **1. Raw Data Processing** | - | + |
| **2. Data Preprocessing** |  |  |
| Missing value imputation | + | + |
| Data filtering | + | + |
| Data normalization | + | + |
| Data transformation | + | + |
| Data scaling | + | + |
| **3. Statistical Analysis** | | |
| Univariate | + | + |
| Multivariate | + | + |
| Clustering | + | + |
| Classification | - | + |
| Complex metadata support | + | + |
| Biomarker analysis | - | + |
| Power analysis | - | + |
| **4. Functional analysis** | - | + |
| **5. Report** | ++ | + |
| **6. Widgets** | | |
| Randomizer | + | - |
| LC tool | + | - |
| MS tool | + | - |
| Metabolite identification | + | ++ |

**Note**:

Symbols used for feature evaluations with ‘+’ for present, ‘-’ for absent, more ‘+’ indicate better support.
