# Supplementary material for: MetaboReport: from metabolomics data analysis to comprehensive reporting
Source: Bioinformatics. 2024 Jun 17;40(6):btae373. doi: 10.1093/bioinformatics/btae373 (PMC11209541; doi:10.1093/bioinformatics/btae373)
Supplement: btae373_Supplementary_Data [file btae373_supplementary_data.zip › FileS1_Manual.pdf]

How to Prepare Input Data for MetaboReport?

2023-02-03

1 Introduction

The use of MetaboReport is pretty simple. You only need to follow the user guide in each tab to perform your data analysis.

MetaboReport accepts a variety of outputs produced by different software tools, such as **XCMS**, **MSDIAL**, and **Compound Discoverer**. The output table can be submitted to MetaboReport with very little modification.

Like any other software tools, MetaboReport requires at least two pieces of information for metabolomics data analysis, i.e., **Peak area** and **sample metadata**.

> `Peak Area`: is the area under a peak (peak area count). It is a measure of the concentration of the peak it represents. Sometimes, **peak intensity** or **peak height** maybe used to represent the the concentration of the peak, depending on the software tools used for data processing.
>
> `Sample Metadata`: or sample label or group label. It is used to describe the sample.

There are **two ways** to prepare input data for MetaboReport:

> **1**. The peak area and metadata are in the same table. You only need to upload one single table for data analysis.
>
> **2**. The peak area table and metadata table are two separated tables. You need to upload both of them for data analysis.

This manual will guide you prepare the input data table for MetaboReport.

2 Data Preparation

The data table can be `csv` (comma-separated values), or MS-Excel format (`xlx`, `xlsx`) format.

2.1 Demo Data

Here an example is used to show you how to prepare the input data table for MetaboReport.

Suppose you want to compare the metabolic profiles between a group of wild type (**WT**) and Mutant (**MU**) mice; `WT` and `MU` are the metadata, meaning each sample is labeled with either a **WT** or **Mu** metadata.

Meanwhile, you believe that gender may also play an important role in your study, so you introduced another set of metadata in your study, namely `Male` and `Female`.

In order to monitor, evaluate and correct system variations in the studies, you also introduced a set of pooled QC (`QC`) samples.

This study contains two groups of metadata, i.e., WT, MU and QC in group 1, and Male, Female and QC in group 2. As such each sample is labeled with two metadata. For instance, Sample3 is WT and Male, and Sample13 is QC and QC (Table 1).

Table 1. Experiment design of a metabolomics study.

Sample	Sample Label 1	Sample Label 2
Sample1	WT	Male
Sample2	WT	Female
Sample3	WT	Male
Sample4	WT	Female
sample5	WT	Male
Sample6	WT	Male
Sample7	MU	Female
Sample8	MU	Male
Sample9	MU	Female
sample10	MU	Male
sample11	MU	Female
sample12	MU	Female
Sample13	QC	QC
sample14	QC	QC
sample15	QC	QC
sample16	QC	QC

There are in total 16 samples in this study (Table 1). After LC-MS analysis and data processing, you will a data table as shown in Table 2. This is a typical sample-feature table, in which each row represents one mass feature, and columns are different descriptors of the feature.

Table 2. An example of sample-feature table.

mz	Retention Time	Identification	PeakArea of Sample1	PeakArea of Sample2	….	PeakArea of Sample16
133.014	3.23	Malic acid	123000	120000	…	801000
144.012	2.44	Unknown	678555	666000	…	1200
333.331	10.18	Unknown	3400	2000	…	560000
234.446	5.52	Unknown	45000	55000	…	46000
…	…	…	…	…	…	…
1034.552	25.33	Tomatine	4780	4300	…	12000

Table 2 will be modified in order to to prepare the required input data table for MetaboReport.

As discuss above, there are two ways to prepare the input data table for MetaboReport.

2.2 Method 1: The peak area and metadata are in the same table.

The method one is simple, you only need to modify **all the peak area columns** shown in Table 2.

For instance, the `PeakArea of Sample1` needs to be modified to `Sample_WT_Male_1`. See Table 3 for details.

If your sample-feature table was generated by Compound Discoverer software, then `PeakArea of Sample1` can be also modified as `WT_Male_1`. The keyword **Sample** is not necessary in this case, but you can keep it as well.

Explanation:

> - Keyword `Sample` need to be added in **Peak Areas** columns are so that MetaboReport will know which columns contain peak area information. Pay attention that the keyword `Sample` is case sensitive.
> - Sample1 has two metadata (see Table 1), so each `metadata`, i.e. **WT** and **MALE**, are added after `Sample` keyword, and they are separated by `_`. Note that `MetaData` are also case sensitive, **WT** and **wt** are different.
> - A `unique number` is added after metadata, which make each sample different from its replicates.
> - all the other columns are not important or necessary for MetaboReport. You don't have to delete them, you can leave them as they are.

Table 3. Sample-Feature table for MetaboReport prepared by method 1.

mz	Retention Time	Sample_WT_Male_1	Sample_WT_Female_2	….	Sample_QC_QC_16
133.014	3.23	123000	120000	…	801000
144.012	2.44	678555	666000	…	1200
333.331	10.18	3400	2000	…	560000
234.446	5.52	45000	55000	…	46000
…	…	…	…	…	…
1034.552	25.33	4780	4300	…	12000

> **Note** that you can also name your raw data in this way before you perform data analysis, then the generated sample-feature table can be directly used by Metaboreport.

2.3 Method 2: The peak area table and metadata table are two separated tables

The second method requires to prepare an additional metadata table.

For **peak area table**:

> - Keyword `Sample` need to be added in **Peak Areas** columns are so that MetaboReport will know which columns contain peak area information. Pay attention that the keyword `Sample` is case sensitive.
> - A `unique number` is needed after the `Sample` Keyword. This is make each sample unique.
> - all the other columns are not important or necessary for MetaboReport. You don't have to delete them, you can leave them as they are.
> - The peak area table can be also prepared exactly in the same way as shown in Mehtod 1.

See Table 4 for details.

Table 4. Peak area table for MetaboReport prepared by method 2.

mz	Retention Time	Identification	Sample1	Sample2	….	Sample16
133.014	3.23	Malic acid	123000	120000	…	801000
144.012	2.44	Unknown	678555	666000	…	1200
333.331	10.18	Unknown	3400	2000	…	560000
234.446	5.52	Unknown	45000	55000	…	46000
…	…	…	…	…	…	…
1034.552	25.33	Tomatine	4780	4300	…	12000

For **Metadata table**:

> You need to prepare a seperate table to descripe the metadata of each sample. See Table 5. The header names are not important. You can give any names, but the first column must be sample. Note that `MetaData` are case sensitive, so pay attention to the consistancy.

Table 5. Metadata table for MetaboReport prepared by method 2.

Sample	Sample Label 1	Sample Label 2
Sample1	WT	Male
Sample2	WT	Female
Sample3	WT	Male
Sample4	WT	Female
sample5	WT	Male
Sample6	WT	Male
Sample7	MU	Female
Sample8	MU	Male
Sample9	MU	Female
sample10	MU	Male
sample11	MU	Female
sample12	MU	Female
Sample13	QC	QC
sample14	QC	QC
sample15	QC	QC
sample16	QC	QC

> **Notice** that: the sample names in Table 4 and Table 5 don't have to be the same, but the samples in peak area table and metadata table must be in the same order; otherwise, wrong metadata will be assigned to the samples. See Figure 2 below.

Figure 1. Pay attention that the samples in peak area table and metadata table must be in the same order.

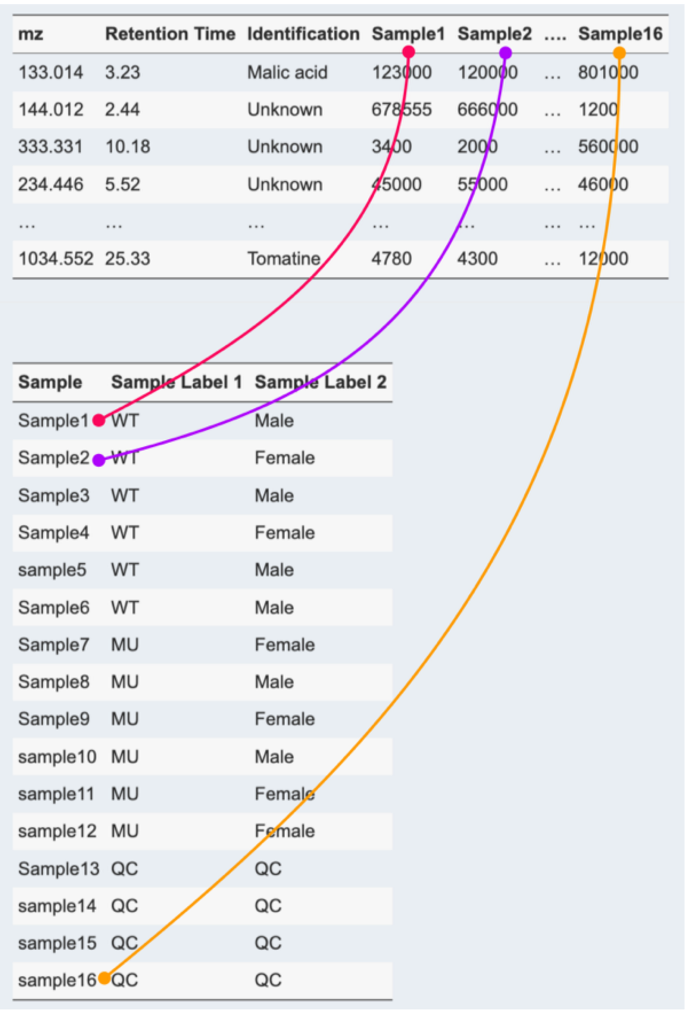

mz	Retention Time	Identification	Sample1	Sample2	….	Sample16
133.014	3.23	Malic acid	123000	120000	…	801000
144.012	2.44	Unknown	678555	666000	…	1200
333.331	10.18	Unknown	3400	2000	…	560000
234.446	5.52	Unknown	45000	55000	…	46000
…	…	…	…	…	…	…
1034.552	25.33	Tomatine	4780	4300	…	12000

Sample	Sample Label 1	Sample Label 2
Sample1	WT	Male
Sample2	WT	Female
Sample3	WT	Male
Sample4	WT	Female
sample5	WT	Male
Sample6	WT	Male
Sample7	MU	Female
Sample8	MU	Male
Sample9	MU	Female
sample10	MU	Male
sample11	MU	Female
sample12	MU	Female
Sample13	QC	QC
sample14	QC	QC
sample15	QC	QC
sample16	QC	QC

3 Demo Data

We have provided two demo datasets, prepared using method 1 and method 2, respectively. Please go to the **Home** Tab of MetaboReport Webapp to download them.

You can refer to the two demo data to help you prepare your own dataset.

4 Common Mistakes

1. My data table is not accepted or not recognized by MetaboReport.

99% of the mistakes are resulted from the inappropriate input data format. Please pay attention to the following points when preparing your data table:

> - Metadata is case sensitive, therefore **WT**, **Wt** and **Wt**, and **wt** will be treated as different metadata, so make sure your metadata are consistent.
> - **Sample** is `reserved keyword`, which is used to help MetaboReport software to recognize **peak area** columns.
> - **QC** is `reserved keyword`, which is used to represent QC samples only.
> - Pay attention to **white spaces**. `WT` and `WT ` with one space will be treated as different metadata.
> - If your input data table is generated by **Compound Discoverer**, please make sure you have exported all the "Area" columns, which contain peak area information; Please also select `Compound Discoverer(CD)`format when uploading the data in **Upload Data** tab. If your data table is produced with other software tools, please select `Other Format`.

2. It takes too long to generate the report.

> - Some additional calculations are being performed during report generation, it may take few minutes to finish.
> - If the report generation is stuck, you may have to restart the PC and re-run your analysis.
